# Similar patient-reported outcomes 6 months after unicompartmental and total knee replacement for osteoarthritis: a prospective cohort study of 60,145 patients from the Australian Orthopaedic Association National Joint Replacement Registry

**DOI:** 10.2340/17453674.2026.46224

**Published:** 2026-07-06

**Authors:** Hannah L FIDLER, George K KIROFF, Peiyao DU, Ilana N ACKERMAN, Helena OAKEY, Peter L LEWIS

**Affiliations:** 1 Faculty of Health and Medical Sciences, University of Adelaide, Adelaide; 2 South Australian Health and Medical Research Institute (SAHMRI), Adelaide; 3 School of Public Health and Preventive Medicine, Monash University, Melbourne; 4 Australian Orthopaedic Association National Joint Replacement Registry (AOANJRR). Adelaide, Australia

## Abstract

**Background and purpose:**

Unicompartmental knee arthroplasty (UKA) is advocated to achieve greater satisfaction and improved function when compared with total knee arthroplasty (TKA), but there is little evidence from large-scale cohorts to support this contention. We aimed to determine, from the patients’ perspective, if UKA leads to postoperative differences in satisfaction, knee pain, function, and quality of life when compared with TKA.

**Methods:**

Data from the Australian Orthopaedic Association National Joint Replacement Registry (AOANJRR) compared PROMs scores for patients with either a UKA or TKA. EQ-5D-5L, Oxford Knee Scores (OKS), and a knee pain rating were recorded preoperatively and 6 months after surgery with additional assessments of satisfaction and patient-perceived change. Comparisons were made for change in mean score after surgery, while percentage change and proportions achieving minimal clinically important change (MCIC) were compared using odds ratios (OR).

**Results:**

3,329 UKAs and 56,816 TKAs were included. UKA patients had higher mean scores for all PROMs measures before surgery, and scores increased similarly to or less than for TKA patients after surgery. Mean OKS was 39 (95% confidence interval [CI] 39–39) after UKA and 38 (CI 38–38) after TKA. Compared with TKA there was a smaller change in mean OKS for the UKA group (–1.3, CI –1.6 to –1.0), which was below the threshold for MCIC. Similar proportions of patients achieved an OKS improvement which met the MCIC threshold. Quality of life improvement after surgery was equivalent for both groups (EQ-5D-5L utility change 0.3 units) as was knee pain improvement (OR 1.1, CI 0.9–1.4) with the UKA group having higher odds of being satisfied (OR 1.2, CI 1.0–1.3) and feeling better (OR 1.12, CI 1.0–1.4), compared with the TKA group.

**Conclusion:**

Based on patient assessments, the difference between UKA and TKA did not achieve the published threshold for a minimal important difference.

Knee osteoarthritis affects approximately 654 million individuals globally [1] and resultant pain, mobility and function restrictions can profoundly impact quality of life (QOL). After exhausting conservative measures, surgery can alleviate severe symptoms. If disease is isolated to the medial compartment, either unicompartmental knee arthroplasty (UKA) or total knee arthroplasty (TKA) may be considered [2]. Some data suggests that UKA may yield superior clinical outcomes in terms of rate of recovery, blood loss, cost, kinematics, and function compared with TKA [3]. However, UKA has almost 3 times the revision rate of TKA at 10 years attributed to progression of osteoarthritis in other knee compartments or loosening of the prosthetic components [4]. Patient-reported outcome measures (PROMs) gauge an individual’s perception of their health, and provide insights into experiences of pain, activity, and QOL [5]. If UKA results in improved function and greater satisfaction, this should be quantifiable by PROMs [3].

While postoperative satisfaction rates may vary, a systematic review found 83% of 112 studies reported satisfaction rates over 80% after TKA [6]. Satisfaction after UKA has been reported as 83% in 1 recent study [7] and 89% in another [8]. Similar rates of post-surgical satisfaction were also found in a recent registry-based survey of TKA and UKA patients in Denmark [9]. However, contemporary studies comparing PROMs from arthroplasty are limited by cohort sizes, restricted prostheses designs and non-standard PROMs measures [10]. National arthroplasty registries allow systematic assessment of arthroplasty outcomes in large populations, reducing many study design shortcomings. Large-scale registry data also offers an opportunity to assess patient outcomes following arthroplasty in relation to meaningful important differences (MID) and the proportion of patients who achieve a minimal clinically important change (MCIC) [11,12].

We aimed to determine, from the patients’ perspective, whether UKA leads to postoperative differences in satisfaction, knee pain, function, and QOL when compared with TKA.

## Methods

### Study design

Data was obtained from the Australian Orthopaedic Association National Joint Replacement Registry (AOANJRR). The AOANJRR commenced data collection on September 1, 1999, achieving complete national implementation by mid-2002. The registry obtains an almost complete dataset (99.2%) of knee replacement performed in Australia [13]. AOANJRR data is externally validated against patient-level data provided by all Australian state and territory health departments. Data is also matched bi-annually with the Australian Government’s National Death Index (NDI) to obtain information on the date of death.

The study was reported according to STROBE guidelines.

### Data

Only patients undergoing primary UKA or TKA with a diagnosis of osteoarthritis who had consented to PROMs score collection were included. Routine demographic and procedure data recorded by the registry (age, sex, body mass index (BMI)—categorized according to the World Health Organization classification, American Society of Anesthesiologists Physical Status classification (ASA) score, type of surgery, and date of surgery)—was matched to electronically collected data obtained from those who elected to participate in AOANJRR PROMs assessments.

### Outcomes

Preoperative and 6-month postoperative PROMs data was collected. Patients who had a revision procedure prior to the postoperative PROMs score collection were excluded. The study period was from the commencement of PROMs collection in July 2018 until December 31, 2023.

Outcomes collected included the EQ-VAS, EQ-5D-5L, Oxford Knee Score (OKS), joint pain, satisfaction, and patient-reported change. The EQ-VAS is a numerical score of health status between 0 and 100 (best) [14]. The EQ-5D-5L is a measure of health status that contains 5 domains (mobility, self-care, usual activities, anxiety/depression, and pain/discomfort) each scored on a 5-point scale. An overall utility score can be calculated using Australian preference weights [15]. A utility score below 0.00 indicates quality of life worse than death and a score of 1.00 indicates full health [16]. The OKS consists of 12 questions that assess knee-related pain and function, producing an overall score of 0 being the poorest function and 48 being the maximal function [17]. Pre- and postoperative affected joint pain experienced over the prior 7 days was scored on a sliding scale from 0 being no pain to 10 being the worst pain imaginable. There were 2 additional postoperative questions. Postoperative satisfaction was rated as very satisfied, satisfied, neutral, dissatisfied, and very dissatisfied. A perceived change question (“How are the problems now with your knee on which you had surgery, compared with before you had your operation?”) was also included. Responses comprised much better, a little better, about the same, a little worse, and much worse.

### Statistics

Linear mixed models for continuous outcomes of EQ-5D-5L, EQ-VAS, and Oxford scores fitted an interaction between time of collection (pre- and postoperative) and group (TKA vs UKA). Confounders age, sex, ASA, and BMI were included in the model as covariates and a random effect for procedure ID allowed for correlation of pre- and postoperative scores from the same procedure. Contrasts were used to calculate mean score changes between pre- and postoperative scores for each group.

Additionally, OKS data was further analyzed against the MCIC, which is the smallest improvement considered worthwhile to a patient. This was done using values previously published by Beard et al. [18]. The change in OKS scores from pre- to postoperative was classified into: ≥ 7 points: minimal clinically important improvement (MCII), < 7 and > 0 points: little improvement, and ≤ 0 points: same or worse. The between-group minimal important difference (MID) in OKS was 5 points as defined by Beard et al. [18]. Log-binomial models adjusting for age, sex, BMI, and ASA score were used for categorical variables (such as estimates of the proportion of patients who were satisfied, achieved a perceived beneficial change, satisfactory pain scores, or attained an MCII) to calculate the event rate. Odds ratios (OR) were used to compare the event rate between groups. We assume missing PROMs are missing at random. A sensitivity analysis in which, for each variable with a missing pre- or postoperative value, the missing value was imputed using the corresponding mean of the available (non-missing) pre- or postoperative values was undertaken.

Statistical analysis was performed using SAS software version 9.4 (SAS Institute Inc, Cary, NC, USA).

### Ethics, registration, data sharing plan, funding, and disclosures

The AOANJRR uses an opt-out consent process for surgical data collection and a separate opt-in consent process for PROMs data collection. The AOANJRR is funded by the Australian Department of Health and Aged Care as a federal quality assurance activity under section 124X of the Health Insurance Act, 1973, and studies are conducted in accordance with ethical principles of research (the Helsinki Declaration II). No funds specific to this project were obtained. There are no conflicts of interest. Complete disclosure of interest forms according to ICMJE are available on the article page, doi: 10.2340/17453674.2026.46224

## Results

**Figure 1:**
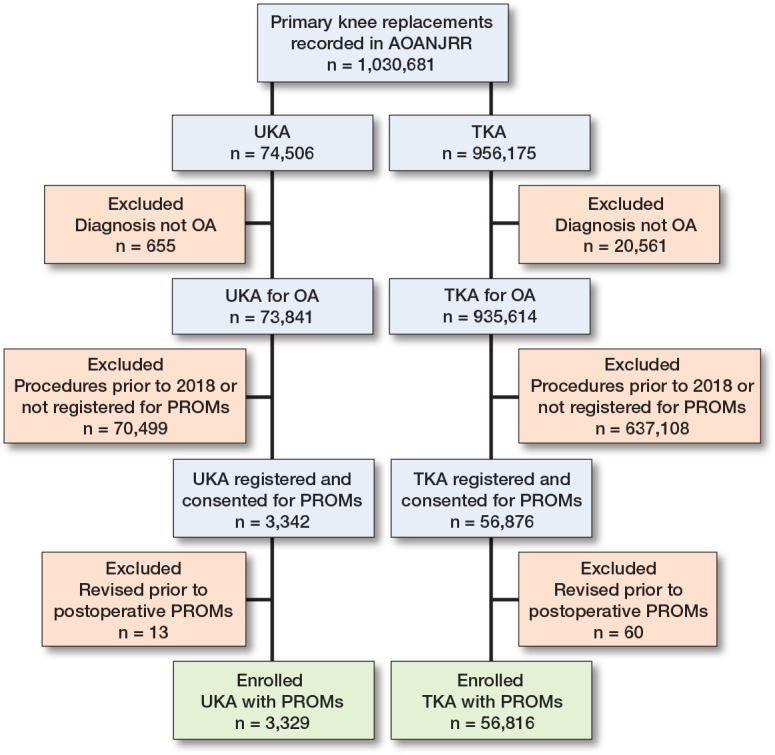
Flow diagram showing summary of study inclusions and exclusions. OA = osteoarthritis; PROM = patient-reported outcome measures; TKA = total knee arthroplasty; UKA = unicompartmental knee arthroplasty.

There were 60,145 arthroplasties enrolled for pre- and postoperative PROMs data collection of which 3,329 and 56,816 were UKAs and TKAs, respectively. Some patients did not respond to the entire suite of questionnaires, which resulted in 1,984 UKA and 32,069 TKA procedures with complete pre- and postoperative PROMs scores, accounting for 57% of those enrolled (Figure 1). The mean age was 68 years for TKA, while patients undergoing UKA were slightly younger (mean age 65 years). Proportionally there were more males in the UKA group (62%) whereas more females had a TKA (55%) (Table 1). The TKA group had a greater proportion of patients with moderate or severe systemic disease (ASA score ≥ 3) (43%) compared with the UKA group (30%). Additionally, patients with obesity (BMI ≥ 30) constituted a larger proportion of the TKA group (59%) compared with the UKA group (44%) (Table 2).

**Table 1:** PROMs assessments for primary knee replacement (KR) by class. Values are count (%)

Variable	UKA	TKA	Total
Primary KR for OA	73,841(7.3)	935,614 (93)	1,009,455
Exclusions			
Prior to 2018	54,242 (77)	616,547 (70)	670,789 (71)
Not registered for			
PROMs	16,257 (23)	262,191 (30)	278,448 (29)
Enrolled in PROMs	3,329 (5.6)	56,816 (94)	60,145
Preoperative completion			
Not completed	325 (9.8)	6,180 (11)	6,505 (11)
Completed	3,004 (90)	50,636 (89)	53,640 (89)
Postoperative completion			
Not completed	1,102 (33)	20,365 (36)	21,467 (36)
Completed	2,227 (67)	36,451 (64)	38,678 (64)
PROMs completion (pre- and postoperative)			
Not completed	1,345 (40)	24,747 (44)	26,092 (43)
Completed	1,984 (60)	32,069 (56)	34,053 (57)

OA = osteoarthritis; TKA = total knee arthroplasty;

UKA = unicompartmental knee arthroplasty.

**Table 2:** Primary knee replacement by class. Values are count (%) unless otherwise specified

Variable	UKA	TKA	Total	Standardized difference
Follow-up, years				
mean (SD)	1.9 (1.4)	1.9 (1.4)	1.9 (1.4)	–0.25
median (IQR)	1.5 (0.8–2.7)	1.5 (0.8–2.8)	1.5 (0.8–2.8)	
range	0–5.4	0–5.4	0–5.4	
Age				
mean (SD)	65 (9.4)	68 (8.6)	68 (8.7)	0.018
median (IQR)	66 (59–72)	68 (62–74)	68 (62–74)	
Age group				
< 65	1,534 (46)	20,117 (35)	21,651 (36)	0.22
≥ 65	1,795 (54)	36,699 (65)	38,494 (64)	
Sex				
Male	2,062 (62)	25,705 (45)	27,767 (46)	0.34
Female	1,267 (38)	31,111 (55)	32,378 (54)	
Procedure year				
2018	102 (3.1)	1,259 (2.2)	1,361 (2.3)	0.050
2019	326 (9.8)	6,359 (11)	6,685 (11)	–0.034
2020	334 (10)	5,711 (10)	6,045 (10)	0.015
2021	597 (18)	9,814 (17)	10,411 (17)	0.016
2022	971 (29)	15,242 (27)	16,213 (27)	0.049
2023	999 (30)	18,431 (32)	19,430 (32)	–0.064
Hospital type				
Public	548 (16)	17,974 (32)	18,522 (31)	
Private	2,781 (84)	38,842 (68)	41,623 (69)	0.37
ASA score ^**a**^				
1	315 (9.5)	2,597 (4.6)	2,912 (4.8)	0.19
2	2,027 (61)	29,918 (53)	31,945 (53)	0.16
3	975 (29)	23,742 (42)	24,717 (41)	–0.25
4	8 (0.2)	481 (0.8)	489 (0.8)	–0.087
5		1 (0)	1 (0)	–0.005
BMI category ^**b**^				
< 18.5	3 (0.1)	98 (0.2)	101 (0.2)	–0.005
18.5–24.9	468 (14)	5,769 (10)	6,237 (10)	0.13
25.0–29.9	1,378 (42)	17,463 (31)	18,841 (32)	0.22
30.0–34.9	974 (30)	17,202 (31)	18,176 (30)	–0.028
35.0–39.9	366 (11)	9,803 (17)	10,169 (17)	–0.18
≥ 40.0	113 (3.4)	6,120 (11)	6,233 (10)	–0.28
BMI group				
< 30.0	1,876 (56)	23,691 (42)	25,567 (43)	
≥ 30.0	1,453 (44)	33,125 (58)	34,578 (57)	0.31
Total	3,329	56,816	60,145	

a American Society of Anesthesiologists Physical Status Classification.

b Body mass index category according to the World Health Organization classification: underweight (< 18.5), normal (18.5–24.9), pre-obese (25.0–29.9), obese Class 1 (30.0–34.9), obese Class 2 (35.0–39.9), obese Class 3 (≥ 40.0).

**Table 3:** Number and (%) of PROMs patients per surgeon

Variable	UKA	TKA	Total	Standardized difference
Patients per surgeon				
1–5	173 (56)	175 (20)	348 (30)	0.80
6–20	89 (29)	180 (21)	269 (23)	0.19
21–50	31 (10)	179 (21)	210 (18)	–0.30
51–100	12 (3.9)	145 (17)	157 (13)	–0.43
> 100	2 (0.6)	189 (22)	191 (16)	–0.71
Total	307	868	1,175	

### Outcomes

**Figure 2:**
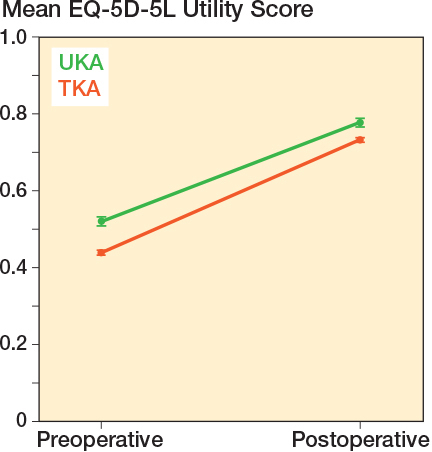
Mean preoperative and postoperative EQ-5D-5L Utility Score in primary knee replacement by class (primary diagnosis osteoarthritis).

Postoperatively, the increase in general health status measured by the EQ-5D-5L Utility Score was the same for both groups, with each having a change in mean score of 0.30 (Figure 2, Table 4). The adjusted mean preoperative score was 0.5 (95% confidence interval [CI] 0.5–0.5) for the UKA group and 0.5 (CI 0.4-0.5) for the TKA group. Correspondingly, when measured by the EQ VAS, the UKA group preoperatively and postoperatively scored higher but there was a larger change in mean score postoperatively for the TKA group (100, CI 9.9–10.2) than for the UKA group (8.0, CI 7.4–8.6) with a difference of –2.0 (CI –2.6 to –1.4) in favor of TKA (Table 4).

**Table 4:** Mean preoperative and postoperative EQ-5D-5L Utility Score, EQ VAS, and OKS in primary knee replacements by class

Class	n	Preoperative		n	Postoperative		Change ^a^ in score (CI)	P value
		Unadjusted mean (SD)	Adjusted ^a^ mean (CI)		Unadjusted mean (SD)	Adjusted ^a^ mean (CI)		
EQ-5D-5L Utility Score UKA	3,073	0.55 (0.26)	0.52 (0.51–0.53)	2,248	0.81 (0.20)	0.78 (0.77–0.79)	0.26 (0.25 to 0.27)	< 0.001
EQ-5D-5L Utility Score TKA	52,253	0.46 (0.31)	0.44 (0.43–0.45)	36,932	0.76 (0.23)	0.73 (0.73–0.74)	0.29 (0.29 to 0.30)	< 0.001
Change: UKA vs TKA							–0.04 (–0.05 to –0.02)	< 0.001
EQ VAS UKA	3,043	74.0 (16.3)	72.2 (71.5–72.3)	2,231	82.4 (14.2)	80.4 (79.7–81.1)	8.2 (7.4 to 8.9)	< 0.001
EQ VAS TKA	51,644	70.1 (18.2)	69.1 (68.7–69.5)	36,524	80.1 (15.4)	78.9 (78.5–79.3)	9.8 (9.6 to 10.0)	< 0.001
Change: UKA vs TKA							–1.6 (–2.4 to –0.9)	< 0.001
OKS UKA	3,038	26.4 (8.0)	25.6 (25.3–25.9)	2,236	40.6 (7.0)	39.6 (39.2–39.9)	14.0 (13.6 to 14.4)	< 0.001
OKA TKA	51,569	23.0 (8.4)	22.6 (22.4–22.8)	36,622	37.9 (7.8)	37.5 (37.3–37.6)	14.8 (14.8 to 14.9)	< 0.001
Change: UKA vs TKA							–0.8 (–1.2 to –0.5)	< 0.001
Affected joint pain UKA	3,033	6.1 (2.1)	6.2 (6.1–6.3)	2,231	1.9 (2.2)	2.0 (1.9–2.1)	–4.2 (–4.3 to –4.1)	< 0.001
Affected joint pain TKA	51,496	6.6 (2.1)	6.6 (6.5–6.6)	36,568	2.4 (2.4)	2.3 (2.3–2.4)	-4.2 (–4.3 to –4.2)	< 0.001
Change: UKA vs TKA							0.01 (–0.1 to 0.1)	0.9

CI = 95% confidence interval; SD = standard deviation.

a Adjusted for age, BMI, sex, and ASA score.

**Figure 3:**
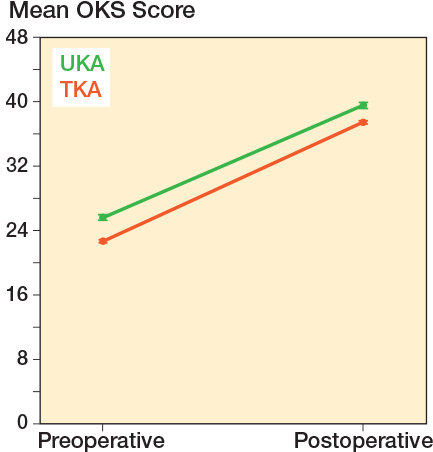
Mean preoperative and postoperative Oxford Knee Score (OKS) in primary knee replacement by class (primary diagnosis osteoarthritis).

Assessments using the OKS also revealed higher pre- and postoperative scores for the UKA group compared with the TKA group (Figure 3). The mean OKS after TKA increased by 15 (CI 15–15) points from 23 to 38. This was comparable to the 14 (CI 13–14) point change after UKA from 26 to 39. A comparison of the change in mean scores favored the TKA group (–1.3, CI –1.6 to –1.0) (Table 4). However, although the between-group comparison is statistically significant the between-group difference is less than the published threshold for an MID [17].

**Figure 4:**
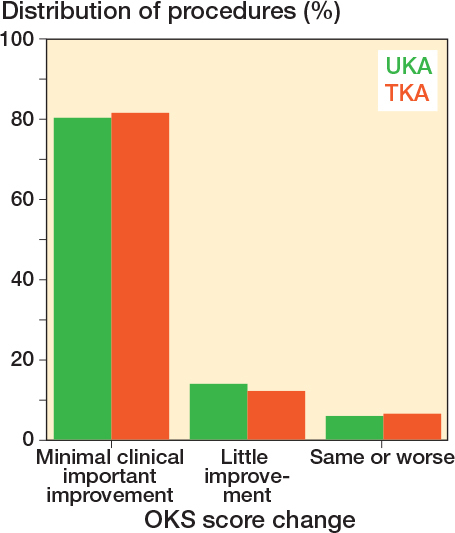
Important change in the Oxford Knee Score (OKS) after primary knee replacements.

An MCII was reached in 80% of UKA patients and in 82% of TKA patients (Figure 4). The arthroplasty groups were similar when assessing the proportions achieving the MCII (OR 1.0, CI 0.9–1.1) (Table 5).

**Table 5:** Postoperative satisfaction, patient-reported change, and affected joint pain in primary knee replacement

Class	Total n	Category	n (proportion)	Rate (CI) ^a^	P value
Satisfaction UKA	2,228	Very satisfied or satisfied	1,937 (0.87)	0.88 (0.86–0.89)	
Satisfaction TKA	36,557	Very satisfied or satisfied	31,174 (0.85)	0.86 (0.85–0.87)	
Odds ratio: UKA vs TKA				1.17 (1.03–1.32)	0.02
Patient-reported change UKA	2,228	Much and a little better	2,078 (0.93)	0.94 (0.93–0.95)	
Patient-reported change TKA	36,550	Much and a little better	33,764 (0.92)	0.93 (0.92–0.94)	
Odds ratio: UKA vs TKA				1.19 (1.00–1.42)	0.046
Affected joint pain UKA	2,002	As or better than before	1,902 (0.95)	0.95 (0.93–0.96)	
Affected joint pain TKA	32,743	As or better than before	30,863 (0.94)	0.94 (0.93–0.95)	
Odds ratio: UKA vs TKA				1.14 (0.93–1.40)	0.2
Oxford Knee Score UKA	2,009	Achieved MCII	1,612 (0.80)	0.82 (0.80–0.84)	
Oxford Knee Score TKA	32,821	Achieved MCII	26,739 (0.82)	0.82 (0.81–0.83)	
Odds ratio: UKA vs TK				1.00 (0.89–1.12)	0.9

CI = 95% confidence interval; MCII = minimal clinically important improvement.

a Adjusted for age, BMI, sex, and ASA score. Proportion is not adjusted.

The affected joint pain scores decreased after both UKA and TKA. For the UKA group the mean score decreased from 6.2 (CI 6.2–6.3) to 2.1 (CI 2.0–2.2) with a change of –4.1 (CI –4.2 to –4.0). The TKA group mean scores decreased from 6.6 (CI 6.5–6.6) to 2.4 (CI 2.3–2.4) with a change of –4.2 (CI –4.3 to –4.2). The change in score was slightly greater in the TKA group (difference 0.1, CI 0.0–0.2) (see Table 4). Postoperatively 94% of UKA patients reported a reduction of knee pain, compared with 94% of TKA patients. 95% of UKA and 94% TKA patients rated their postoperative affected joint pain score as better than preoperatively (OR 1.1, CI 0.9–1.4) (see Table 5).

When asked about satisfaction with their knee procedure, there was a slightly larger proportion of satisfied patients in the UKA group (combined satisfied/very satisfied 88%, CI 86–89 for UKA and 86%, CI 85–87 for TKR; OR 1.2, CI 1.0–1.3) (Table 5). Both UKA and TKR groups had 2% who were very dissatisfied with the outcome.

Most patients reported feeling much better after surgery (85% of UKA and 82% of TKA). The proportion reporting better outcomes (combining the much better and a little better groups) was 94% for UKA and 93% for TKA patients. The comparison of patient reported change slightly favored the UKA group (OR 1.2, CI 1.0–1.4) (Table 5).

We found no material differences when comparing results with the sensitivity analysis (Supplementary data). This suggests that our results are robust due to our large sample size.

## Discussion

We aimed to determine, from the patients’ perspective, if UKA leads to postoperative differences in satisfaction, knee pain, function, and QOL when compared with TKA.

We found very little difference between the groups when comparing UKA with TKA outcomes from the patients’ perspective. When compared with TKA patients using PROMs, UKA patients feel slightly better, are marginally more likely to be satisfied, but show less improvement in pain score, QOL, and knee function as measured by OKS after surgery. While some of these comparisons achieve statistical significance this does not equate to a clinical difference. When comparing these groups by the magnitude of change in OKS there was no meaningful important difference, and similar proportions achieved an MCII.

There was a similar prevalence of postoperative satisfaction scores after UKA and TKA procedures (87% and 85% respectively). This between-group similarity is further supported when comparing knee pain scores. The change in QOL measured by the EQ-5D-5L Utility Score was equivalent, but the EQ-VAS assessments favored the TKA group, while patient-reported change score percentage comparisons were slightly greater following UKA procedures. There was no clear pattern of better outcomes for 1 procedure type across the suite of instruments.

Meta-analyses and systematic reviews comparing the 2 treatments have shown mixed results and have been unable to determine a clearly superior option [19–21]. A study by Annapareddy et al. specifically using PROMs to compare the 2 surgical options also reported similar results, reporting a marginally higher satisfaction rate in UKA patients, with no significant differences observed in the mean improvement of other PROMs [22]. These results contrast with observational studies from the Oxford group, which demonstrated greater proportions of excellent outcomes and satisfaction for the UKA group [10,23]. Our results are also similar to those from a large, randomized trial [23] and previous registry studies comparing UKA and TKA [9, 24, 25]. These prior studies had not assessed meaningful difference or clinically important change in OKS.

### Strengths

The strengths of our comparison study include the large patient cohort, use of standardized PROMs instruments, and exploration of PROMs scores through the context of MCIC.

### Limitations

There is likely some selection bias, precluding those with the most severe joint disease, as patients with tricompartmental OA would not be considered for a UKA. Unfortunately, the registry does not capture details of the extent of preoperative disease; however, where possible we adjusted for known confounders. Less severe OA and preoperatively better-functioning knees most likely make up a greater proportion of the UKA group who were younger, more often male, and had higher preoperative PROMs scores. A high preoperative score decreases the potential for improvement measured by PROMs and ceiling effects may be pertinent for some of the instruments. The ceiling effect was seen particularly postoperatively for the EQ-5D-5L Utility Score where the upper quartile limit for UKA patients postoperatively reached the maximum score. Where high postoperative scores are common it becomes difficult to distinguish those who are very good from those who are very, very good. This constitutes a constraint to measuring the actual improvement statistically. It is possible that a difference may be found if other PROMs instruments, such as the Forgotten Joint Score [26], were chosen, as recently suggested by Bayram et al. [27].

While the ideal time for collecting postoperative information is debated, it is thought that 6 months allows for much of the postoperative improvement to have occurred while maintaining adequate capture rates [28]. However, this time point may favor UKA patients, who tend to recover more quickly than those with a TKA, and different results might have been found after 2 years. A further limitation may be the exclusion of patients who required revision surgery before 6 months. While this number is very small (73 patients), it is expected that revised patients would have had low postoperative PROMs scores and, if included, these patients may have decreased the derived mean postoperative scores. Also, as revision is more common among UKA patients [4], this may have unequally affected the study groups.

We recognize the limitations of statistical testing, particularly when comparing mean scores for skewed populations. The rate of revision is a traditional and clearly defined way to determine a poor outcome from surgery [29]. Discussion regarding the superiority of TKA over UKA is largely based on the comparison of revision rates [30,31] while the supporters of UKA cite more difficult to measure factors such as better function, speed of recovery, and improved joint proprioception [10,32], or increased proportions who return to work [33]. However, we resolved that more meaningful analyses are made comparing the proportions achieving the MCIC and using the MID as suggested by Beard et al. [17].

Finally, not all knee arthroplasty patients complete PROMs, and while this may not be indicative of the entire knee replacement population, a previous report from the AOANJRR by Harris et al. in 2021 [34] has shown that electronic responders to PROMs are likely to represent the overall cohort. Efforts to further expand national PROMs collection and improve capture rates are ongoing.

### Conclusion

Based on patient assessments, there are similar UKA and TKA outcomes, and high rates of satisfaction were achieved for both procedures. These findings can be used to counsel patients and inform shared decision-making.

### Supplementary data

Supplementary Tables 1–3 are available as supplementary data on the article page, doi: 10.2340/17453674.2026.46224

## Supplementary Material



## Notes

HF: Study design, literature review, data interpretation, manuscript preparation. GK: Study design, methodology advice, manuscript preparation. PD: Data provision, data analysis. IA: Study design, methodology advice, data interpretation, manuscript preparation. HO: Data provision, data analysis and interpretation, manuscript preparation. PL: Study conceptualization, study design, data interpretation, manuscript preparation

Handling co-editors: Per Henrik Randsborg and Robin Christensen

*Acta* thanks Ove Nord Furnes and other anonymous reviewers for help with peer review of this manuscript.
