# Supplementary material for: Similar patient-reported outcomes 6 months after unicompartmental and total knee replacement for osteoarthritis: a prospective cohort study of 60,145 patients from the Australian Orthopaedic Association National Joint Replacement Registry
Source: Acta Orthop. 2026 Jul 6;97:471–7. doi: 10.2340/17453674.2026.46224 (PMC13334313; doi:10.2340/17453674.2026.46224)
Supplement: Supplementary file 1 [file ActaO-97-46224-s1.pdf]

Supplementary data

Sensitivity analysis

Table 1. Summary of missing data. Values are count (%)

Variable		UKA	TKA	TOTAL
Preoperative completion				
	Not completed	325 (9.8)	6,180 (11)	6,505 (11)
	Completed	3,004 (90)	50,636 (89)	53,640 (89)
Postoperative completion				
	Not completed	1,102 (33)	20,365 (36)	21,467 (36)
	Completed	2,227 (67)	36,451 (64)	38,678 (64)
PROMs completion (pre- and postoperative)				
	Not completed	1,345 (40)	24,747 (44)	26,092 (43)
	Completed	1,984 (60)	32,069 (56)	34,053 (57)
Preoperative EQ-5D-5L Utility completion				
	Not completed	36 (1.1)	1,013 (1.8)	1,049 (1.7)
	Completed	3,293 (99)	55,803 (98)	59,096 (98)
Postoperative EQ-5D-5L Utility completion				
	Not completed	36 (1.1)	1,013 (1.8)	1,049 (1.7)
	Completed	3,293 (99)	55,803 (98)	59,096 (98)
Preoperative EQ VAS Health completion				
	Not completed	57 (1.7)	1,379 (2.4)	1,436 (2.4)
	Completed	3,272 (98)	55,437 (98)	58,709 (98)
Postoperative OKS completion				
	Not completed	64 (1.9)	1,446 (2.5)	1,510 (2.5)
	Completed	3,265 (98)	55,370 (98)	58,635 (98)
Preoperative Affected Joint Pain completion				
	Not completed	67 (2.0)	1,495 (2.6)	1,562 (2.6)
	Completed	3,262 (98)	55,321 (97)	58,583 (97)
Postoperative Affected Joint Pain completion				
	Not completed	67 (2.0)	1,495 (2.6)	1,562 (2.6)
	Completed	3,262 (98)	55,321 (97)	58,583 (97)
Procedure Satisfaction completion				
	Not completed	1,101 (33)	20,259 (36)	21,360 (35)
	Completed	2,228 (67)	36,557 (64)	38,785 (65)
Patient Reported Change completion				
	Not completed	1,101 (33)	20,266 (36)	21,367 (35)
	Completed	2,228 (67)	36,550 (64)	38,778 (65)
TOTAL		3,329	56,816	60,145

Table 2. Mean preoperative and postoperative EQ-5D-5L Utility Score, EQ VAS and OKS in primary knee replacement by Class (sensitivity analysis)

	Preoperative		Postoperative			
Class	n	mean (CI)	n	mean (CI)	Change in score	P-value
EQ-5D-5L Utility Score UKA	3,329	0.52 (0.51–0.54)	3,329	0.78 (0.77–0.78)	0.25 (0.24 to 0.26)	<0.001
EQ-5D-5L Utility Score TKA	56,816	0.45 (0.44–0.45)	56,816	0.74 (0.74–0.75)	0.30 (0.29 to 0.30)	<0.001
Change: UKA vs TKA					–0.05 (–0.06 to –0.04)	<0.001
EQ VAS UKA	3,329	72.5 (71.9–73.1)	3,329	80.5 (80.1–81.0)	8.0 (7.4 to 8.6)	<0.001
EQ VAS TKA	56,816	69.6 (69.3–69.8)	56,816	79.6 (79.3–79.8)	10.0 (9.9 to 10.2)	<0.001
Change: UKA vs TKA					-2.0 (-2.6 to -1.4)	<0.001
OKS UKA	3,329	25.6 (25.3–25.9)	3,329	39.2 (38.9–39.4)	13.6 (13.3 to 13.9)	<0.001
OKA TKA	56,816	22.8 (22.6–22.9)	56,816	37.7 (37.6–37.9)	15.0 (14.9 to 15.0)	<0.001
Change: UKA vs TKA					–1.3 (–1.6 to –1.0)	<0.001
Affected Joint Pain UKA	3,329	6.2 (6.2–6.3)	3,329	2.1 (2.0–2.2)	–4.1 (–4.2 to –4.0)	<0.001
Affected Joint Pain TKA	56,816	6.6 (6.5–6.6)	56,816	2.4 (2.3–2.4)	–4.2 (–4.3 to –4.2)	<0.001
Change: UKA vs TKA					0.11 (0.02 to 0.20)	0.02

Table 3. Postoperative satisfaction, patient reported change and affected joint pain in primary knee replacement (sensitivity analysis)

Class	Total n	Category	Rate (CI)	P value
Satisfaction UKA	3,329	Very satisfied or satisfied, n = 3,038	0.92 (0.91–0.93)	
Satisfaction TKA	56,816	Very satisfied or satisfied, n = 51,433	0.91 (0.90–0.92)	
Odds ratio: UKA vs TKA			1.12 (0.99–1.27)	0.08
Patient reported change UKA	3,329	Much and a little better, n = 3,179	0.96 (0.95–0.97)	
Patient reported change TKA	56,816	Much and a little better, n = 54,030	0.95 (0.95–0.96)	
Odds ratio: UKA vs TKA			1.15 (0.97–1.36)	0.1
Affected Joint Pain UKA	3,329	As or better than before, n = 1,124	0.34 (0.32–0.36)	
Affected Joint Pain TKA	56,816	As or better than before, n = 18,214	0.32 (0.31–0.33)	
Odds ratio: UKA vs TKA			1.08 (1.00–1.16)	0.052
Oxford Knee Score UKA	3,329	Achieved MCII, n = 2,932	0.89 (0.88–0.91)	
Oxford Knee Score TKA	56,816	Achieved MCII, n = 50,734	0.90 (0.89–0.91)	
Odds ratio: UKA vs TK			0.97 (0.87–1.08)	0.5

MCII = Minimal clinically important improvement
